# It's not what you say but the way that you say it: an fMRI study of differential lexical and non-lexical prosodic pitch processing

**DOI:** 10.1186/1471-2202-12-128

**Published:** 2011-12-20

**Authors:** Derek K Tracy, David K Ho, Owen O'Daly, Panayiota Michalopoulou, Lisa C Lloyd, Eleanor Dimond, Kazunori Matsumoto, Sukhwinder S Shergill

**Affiliations:** 1 CSI Lab, Institute of Psychiatry, King's College London, UK; 2 Department of Psychiatry, Tohoku University School of Medicine, Sendai, Japan

## Abstract

**Background:**

This study aims to identify the neural substrate involved in prosodic pitch processing. Functional magnetic resonance imaging was used to test the premise that prosody pitch processing is primarily subserved by the right cortical hemisphere.

Two experimental paradigms were used, firstly pairs of spoken sentences, where the only variation was a single internal phrase pitch change, and secondly, a matched condition utilizing pitch changes within analogous tone-sequence phrases. This removed the potential confounder of lexical evaluation. fMRI images were obtained using these paradigms.

**Results:**

Activation was significantly greater within the right frontal and temporal cortices during the tone-sequence stimuli relative to the sentence stimuli.

**Conclusion:**

This study showed that pitch changes, stripped of lexical information, are mainly processed by the right cerebral hemisphere, whilst the processing of analogous, matched, lexical pitch change is preferentially left sided. These findings, showing hemispherical differentiation of processing based on stimulus complexity, are in accord with a 'task dependent' hypothesis of pitch processing.

## Background

Non-verbal components of language, included under the collective term prosody, play a central role in human communication [1]. First defined by Monrad-Krohn in 1947 [2], prosodic elements of speech can be subdivided into the two broad categories of linguistic and emotional prosody. Linguistic prosody conveys information about semantic meaning, such as pragmatic category - e.g. determining if a sentence is a statement, a question or a command - and syntactic relation - e.g. determining clause boundaries within sentences [3,4]. Emotional prosody is the mechanism by which humans convey attitudes and emotions in speech. There has been debate about how clearly these two categories can be delineated.

Initial behavioural and lesion studies implicated both right [5-9] and left [10-12] hemispheric regions, likely confounded both by the inherent difficulties in comparing lesion studies [13,14] and assessing "global" prosodic function without considering specific subcomponents.

PET data first suggested that prosodic content and judgement activated the prefrontal cortex bilaterally [15,16], more so on the left, and hemispheric asymmetry has been demonstrated for most regions of activation [17]. Subsequent imaging studies have implicated right superior temporal regions [16,18-21] - most recent work suggesting particularly within Brodmann's area [22], with additional, partially bilateral responses within the frontal cortex [18,20,23-25], the anterior insula [16,23,25], amygdalae [26], and the basal ganglia [27,28]. Emotional speech produces greater cortical activation than that which is prosodically neutral [16,22,29]. Electrophysiological work has supported neuroimaging findings that the right temporal cortex displays enhanced event-related potentials to emotional stimuli [30].

Variations in results, due in no small part to different experimental paradigms, have failed to definitively clarify whether cerebral regional and hemispheric activation are specific to prosodic subcomponent analysis or the functional demand of the task, known as the *cue dependent *[31,32] and *task dependent *[17,33-36] hypotheses respectively.

However by far the majority of work has been on emotional prosody, and it's unclear how well such data can be applied to linguistic prosody that, in comparison, has had a paucity of research. Furthermore, work on linguistic or semantic aspects of prosody have typically focused on psychometric measures of language conceptualisation and understanding [37,38] rather than the underlying neurobiology. Most authors have recognized the difficulties of the confounding influences of the lexical content of the stimuli and the problem of the higher level cognitive processes involved in the more global process of emotional prosody [39].

The neuroimaging data that exist for linguistic prosody typically favour hemispheric specialisation [40], with left fronto-temporal regions subserving 'simpler' short [41] syntactic and lexical segments of speech [42], and right hemispheric analogues processing larger suprasegmental elements at a sentence level [43], most in keeping with the task dependent hypothesis.

In light of this, this study set out to utilise fMRI to examine a single crucial element of linguistic prosodic comprehension; pitch change. We specifically looked at *internal pitch changes*, or "emphasis shift", as our earlier work suggested that these were more sensitive markers of subtle neurological deficits and less confounded by working memory primacy and recency phenomena [44]. As the name suggests, internal pitch changes occur *within *- as opposed to at the beginning or end of - a sentence. Furthermore, in an effort to try eliminate the major confounder of lexical comprehension, following the work of Patel et al [45] we introduced an analogous tone-sequence paradigm that contained a delexicalised pitch pattern. By removing the lexical content but keeping the tone sequence otherwise matched this design would also allow testing of the validity of the task dependent hypothesis as the same prosodic element, pitch, was being tested, but at different levels, with the tone sequence involving suprasegmental data analysis.

We hypothesised that a) there are common cortical regions including bilateral prefrontal and temporal cortices associated with pitch processing in both speech and tone-sequence analogues; b) the more "pure" pitch processing associated with tone-sequence analogues would preferentially recruit right sided frontal and temporal cortices while more lexically loaded speech would preferentially recruit left temporal cortex; and c) increasing demands on prosodic comprehension would be associated with enhanced activation in the right frontal and temporal cortex.

## Methods

### Subjects

Twelve subjects were recruited through advertisements in a city-wide newspaper. Inclusion criteria were: males aged between 18 and 55, right-handedness, English as a first language. Exclusion criteria were: previous psychiatric or neurological illness, hearing or speech impairment and illicit drug use in the previous six months. All subjects provided written informed consent. Mean age was 31 (SD = 9.6). All subjects had completed secondary education; none had any formal training in playing musical instruments. The study had been approved by the local ethics committee.

### Stimuli and materials

A modified version of the tone-sequence and prosody discrimination task previously described by the authors [44] was used, based on the earlier protocol of Patel et al [45]. The recorded stimuli consisted of 12 lexically identical sentence pairs, spoken by an adult female native English speaker, and their non-verbal, tone-sequence analogue pairs; and 12 sentence and tone-sequence pairs that differed prosodically in internal pitch pattern on a single word or tone (e.g. "I like blue ties on gentlemen" and "I like *blue *ties on gentlemen", with the italicized word emphasised). Tone-sequence stimuli were created by digitizing each sentence at 40,000 Hz with subsequent normalization to the same amplitude into a tone sequence which corresponded with the sentence's fundamental frequency in pitch and timing, with one level-pitch tone per syllable; a more detailed description is available in Patel et al [45]. An alternative method of low-pass filtering of the sentence pairs to remove lexical information was felt to be less satisfactory, as such filtering can leave residual phonological information, and previous studies [46] had validated this method.

### Procedure

Subjects were trained on the prosodic discrimination task, which consisted of six counterbalanced blocks. Each block was composed of twelve trials comprising four pairs of sentences, four pairs of tone-sequences, and four null trials (a silent period equal in length to four paired stimuli) presented in random order. Each trial consisted of a pair of stimuli separated by a one second interval. The pair of stimuli differed in the pitch of an internal component in 50% of trials. As some sentences were longer than others, the duration of the stimuli varied from 3432-6134 milliseconds, with an average length of 5036 ms. Following a visual cue at the end of each trial, subjects indicated whether the paired stimuli were the same or different by using their right index finger and a button press. There was a variable intertrial interval of between 8.6-11.3 seconds before the onset of the next trial. Such a jittered design results in peristimulus distribution of MRI sampling, thus ensuring that all components of an event-related haemodynamic response are sampled, and avoids that bias of having stimulus presentation and data acquisition time-locked [46]. The total length of the six counterbalanced blocks was 17 minutes 39 seconds.

### fMRI Acquisition

Gradient echo echoplanar imaging (EPI) data were acquired on a neuro-optimised GE Signa 1.5 Tesla system (General Electric, Milwaukee WI, USA) at the Maudsley Hospital, London. A quadrature birdcage headcoil was used for radio frequency transmission and reception. Foam padding was placed around the subject's head in the coil to minimize head movement. One hundred and forty four T2*-weighted whole-brain volumes depicting blood oxygen level-dependent (BOLD) contrast were acquired at each of 24 near-axial non-contiguous planes parallel to the intercommissural (AC-PC) line (slice thickness = 5 mm; gap = 0.5 mm; TR = 2.1 seconds; echo time = 40 milliseconds; flip angle = 90°; matrix = 64 × 64). This EPI data set provided complete brain coverage. At the same session, a high-resolution gradient echo image of the whole brain was acquired in the intercommissural plane consisting of 43 slices (slice thickness = 3 mm; gap = 0.3 mm; TR = 3 seconds; flip angle = 90°; matrix = 128 × 128).

Scanner noise during stimuli presentation was minimised by using a partially silent acquisition [47] during the stimuli presentation lasting 6.3 seconds while fMRI data (associated with prominent scanner noise) was collected during the following 8.4 seconds.

### fMRI Analysis

The data were first realigned [48] to minimise motion related artefacts and smoothed using a Gaussian filter (FWHM 7.2 mm). Responses to the experimental paradigm were then detected by time-series analysis using Gamma variate functions (peak responses at 4 and 8 sec) to model the BOLD response. The analysis was implemented as follows. First, in each experimental condition, trial onsets were modelled as stick-functions which were convolved separately with the 4 and 8 sec Poisson functions to yield two regressors of the expected haemodynamic response to that condition. The weighted sum of these two convolutions that gave the best fit (least-squares) to the time series at each voxel was then computed and a goodness of fit statistic was computed at each voxel, the SSQratio. It has been shown that this permutation method gives very good type I error control with minimal distributional assumptions [49].

In order to extend inference to the group level, the observed and randomized SSQratio maps were transformed into standard space by a two stage process involving first a rigid body transformation of the fMRI data into a high-resolution inversion recovery image of the same subject followed by an affine transformation onto a Talairach template [50]. In order to increase sensitivity and reduce the multiple comparison problem encountered in fMRI, hypothesis testing was carried out at the cluster level using the method developed by Bullmore et al. [48], shown to give excellent cluster-wise type I error control in functional fMRI analysis. All analyses were performed with < 1 false positive clusters expected per image, under the null hypothesis.

We examined regions of activation common to both sentence and tone-sequence prosodic comprehension with conjunction analysis. As the levels of activation in the various experiments will vary, the statistical issue is whether the minimum level of activation in any of the tasks is significantly different from zero. In parametric analysis this is done by testing the minimum t statistic. The statistical analysis program utilized (XBAM) found which task had the smallest median level of activation and tested this median against the null distribution of the activation by estimating the SSQratio for each subject at each voxel for each task [49,51]. Then we compared prosodic comprehension between the tone-sequence and sentence stimuli to clarify the effects of lexical processing. Subsequent analyses compared identical stimuli pairs with differing stimuli pairs (same versus different stimuli pairs). During the pilot phase, volunteers subjectively reported the appraisal of identical stimuli to be more demanding. This was used to examine the effects of postulated increased demand on prosodic assessment. We employed a 2 × 2 factorial design to examine the interaction of factor condition (tone sequence, sentence) with the variables of pair type (same, different). The SSQ values were extracted from whole clusters, and plotted for regions demonstrating significant interaction effects between tone sequence and sentence processing and task demand assessed by same or differing stimuli pairs.

A confounder in all fMRI studies is the intrinsic scanner noise: this is particularly the case in tasks with an auditory component such as this one. We minimized this by having the scanner at a partially silent acquisition phase [47] during the presentation of stimuli. It has been shown that handedness and gender may affect the neural structures involved in the processing of language [52] and prosody [53], as such we only examined right handed males.

Behavioural data were analyzed using the statistical package SPSS.

## Results

### Behavioural data

There were no significant differences in response time or accuracy rates between sentence and tone-sequence categories either overall, or when analysed in the subcategories of *same *and *different *tasks, using a two tailed t-test (α = 0.05). The subjects were generally highly accurate (0.75 - 0.98 on the tone-sequence task, 0.83 - 1.00 on the sentence task), with four individuals getting 100% accuracy on the sentence task, suggestive of a possible ceiling effect. However, subjects were more accurate during *same *tasks (mean accuracy 0.948) than during *different *tasks (mean accuracy 0.866) overall.

### Neuroimaging data

**Figure 1:**
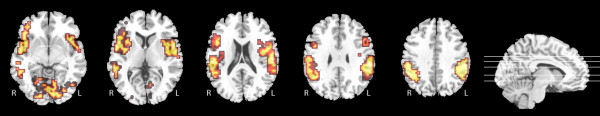
**Conjuction analysis of regions of cerebral activation common to both the sentence and tone-sequence tasks**. 5 ascending transverse slices, with a sagittal section to the right of the image indicating where these are taken from. Exact cluster coordinates are given in Table 1.

The conjunction analysis showed significant activation common to both sentence and tone sequence prosodic processing in the bilateral Inferior Frontal Gyri, Middle (MTG) and Superior Temporal Gyri (STG), in addition to bilateral Inferior Parietal lobule and the right Superior Frontal Gyrus (Figure 1; Table 1).

**Table 1:** Areas of activation shown in *Figure 1*.

Size	Talairach Coordinates			Hem	BA	Cerebral Region
	X	Y	Z			
62	-43	-33	48	L	40	Inferior Parietal Lobule

60	-54	-22	37	L	2	Postcentral Gyrus

48	51	7	-7	R	22	Superior Temporal Gyrus

43	-54	-15	9	L	41	Middle Temporal Gyrus

42	47	-48	26	R	40	Inferior Parietal Lobule

40	-54	0	-2	L	22	Superior Temporal Gyrus

32	-32	-26	59	L	4	Precentral Gyrus

32	-7	-81	-13	L	18	Lingual Gyrus, Occipital Lobe

30	51	15	-2	R	47	Inferior Frontal Gyrus

12	58	-37	-7	R	21	Middle Temporal Gyrus

10	32	15	4	R		Claustrum

Hem = hemisphere, BA = Brodmann's Area.

**Figure 2:**
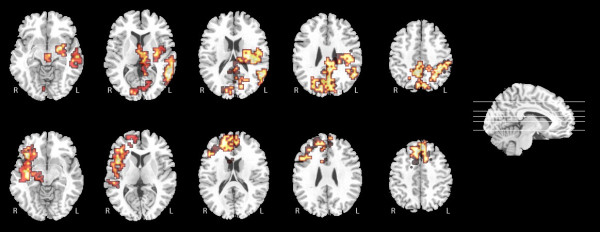
**ANOVA of regions of task-dependent differential activation**. Ascending transverse slices with the sagittal section to the right indicating where they are taken from. The top half displays regions of relative increased activation during the sentence task; the lower half displays those more active during the tone-sequence task. The exact cluster coordinates are provided in Table 2 and Table 3 respectively.

Activation was significantly greater within the right frontal and temporal cortices during the tone-sequence stimuli relative to the sentence stimuli (Figure 2, bottom half; Table 2). Regions of greater activation in the sentence task relative to the tone sequence task (Figure 2, top half; Table 3) were predominantly left hemispheric, including the cingulate gyrus, left MTG, STG, inferior parietal lobule as well as the basal ganglia; with additional activation in the right precuneus, right cingulate gyrus and right lingual gyrus.

**Table 2:** Areas of activation shown in *Figure 2, BOTTOM HALF*.

Size	Talairach Coordinates			Hem	BA	Cerebral Region
	X	Y	Z			
71	32	30	-13	R	47	Inferior Frontal Gyrus

41	7	19	42	R	32	Cingulate Gyrus, Limbic Lobe

40	11	41	37	R	7	Medial Frontal Gyrus

29	51	11	-2	R	22	Superior Temporal Gyrus

22	36	22	4	R	13	Insula

17	14	11	53	R	6	Superior Frontal Gyrus

10	18	-19	-18	R	28	Parahippocampal Gyrus

9	-7	4	53	L	6	Medial Frontal Gyrus

Hem = hemisphere, BA = Brodmann's Area.

**Table 3:** Areas of activation shown in *Figure 2-TOP HALF*.

Size	Talairach Coordinates			Hem	BA	Cerebral Region
	X	Y	Z			
97	-14	-48	26	L	31	Cingulate Gyrus, Limbic Lobe

58	7	-63	31	R	7	Precuneus

47	-29	-11	9	L		Putamen

44	-54	-19	-7	L	21	Middle Temporal Gyrus

32	-47	-26	37	L	2	Postcentral Gyrus

27	-18	-22	15	L		Posterior Thalamic Nucleus

26	4	-44	37	R	31	Cingulate Gyrus, Limbic Lobe

24	14	-78	4	R	18	Lingual Gyrus, Occipital Lobe

19	-54	-52	9	L	39	Superior Temporal Gyrus

18	-29	-4	-13	L		Amygdala

15	-22	-56	42	L	7	Precuneus

12	-40	-33	26	L	40	Inferior Parietal Lobule

Hem = hemisphere, BA = Brodmann's Area.

**Figure 3:**
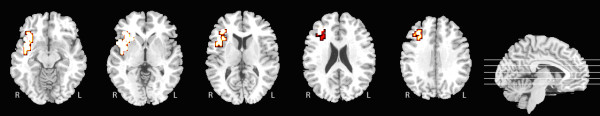
**Interaction analysis of cerebral regions that can differentiate task (sentence/tone-sequence) and trial type (same/different)**. Cluster coordinates are provided in Table 4.

A statistically significant interaction between *factor *condition (tone sequence, sentence) and stimulus pair *type *(same, different) was evident in the right Inferior and Middle Frontal Gyrus and right STG (Figures 3; Table 4).

**Table 4:** Areas of activation in *Figure 3*.

Size	Talairach Coordinates			Hem	BA	Cerebral Region
	X	Y	Z			
21	43	7	-13	R	38	Superior Temporal Gyrus

11	43	15	-2	R	47	Inferior Frontal Gyrus

9	36	33	20	R	46	Middle Frontal Gyrus

Hem = hemisphere, BA = Brodmann's Area.

## Discussion and Conclusions

As hypothesized, there was activation in bilateral MTG and STG common to prosodic pitch processing across both sentence and tone-sequence stimuli. There was more prominent right inferior frontal cortex activation, although left inferior frontal activation was also present (Figure 1; Table 1). This was in accordance with previous imaging data of prosodic comprehension [18,20,23-25]. There was a large bilateral activation in the Inferior Parietal Lobule, a region associated with storage within the working memory system [1]. Such a role is in accordance with our data, as differential activation maps fail to show differences in parietal activation between the two tasks, coinciding with a purely working memory role. Left precentral and postcentral gyral and basal ganglia activity were common to both conditions, something which would be anticipated in an experimental paradigm involving a right handed finger press.

Comparison between the tone-sequence and sentence stimuli aimed to clarify the relative contribution of cortical regions associated with a purer linguistic prosodic pitch analysis (tone sequence > sentences) and those associated with greater lexical or phonological analysis (sentence > tone sequence), which has been recognised as a major confounder in such studies generally [45,54-59]. Stripped of this lexical information, the tone-sequence demonstrated significant activation in the *right *inferior and medial frontal, and right STG compared to the sentence task (Figure 2, bottom half; Table 2). Wildgruber et al [1] suggested that at lower levels of both linguistic and emotional prosody processing, the same right hemispheric network is accessed but that the explicit judgment of linguistic aspects of speech prosody is more associated with left hemispheric language regions and explicit evaluation of emotional prosody is related to bilateral orbitofrontal regions. Our data support this assertion, with evident overlap between the regions preferentially activated by the tone-sequence and those elicited during emotional prosodic tasks. Explicit analysis of linguistic aspects preferentially evoked appraisal by left hemispheric regions, fitting with other work [34,55,60-62] and this may reflect the processing of this lexical content of the stimuli.

Our third hypothesis was that 'increased demand' would be associated with enhanced activation in the right frontal and temporal cortices. Subjects reported finding tone-sequence trials harder than sentence ones - fitting with Patel's notion of extra 'redundancy' cues in the lexical trials [45,58] - and that *same *pairs were 'more difficult' than *different *ones. Interestingly, behavioural data conflicts with subjective perception, demonstrating that subjects more accurate in *same *tasks: during these subjects needed to hold the entire *same *trial pair in working memory, and examine these for subtle (non-existent) differences; as opposed to *different *pairs where participants could discard the stimuli once *any *pitch difference was noted. As such, *same *and *tone-sequence *may have been proxy markers for cognitive demand rather than 'difficulty' per se, as well as tone-sequence exploring 'purer' pitch processing.

**Figure 4:**
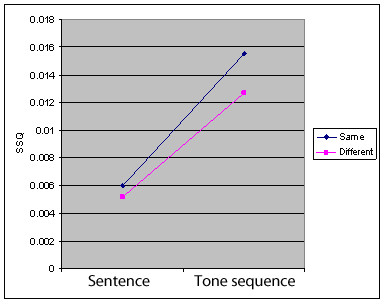
**Graphed differential activation in the right inferior frontal gyrus in the interaction analysis of Figure 3 demonstrating activation in the two task types (sentence/tone-sequence) and two trial types (same/different)**. SSQ, a "sum of squares ratio" is a statistical indicator of activity, as described in the methods section.

During the tone-sequence task there was *relatively *increased right STG and left precuneus activation when paired tone stimuli were the *same*, compared to when different. The interaction analysis (Figures 3; Table 4) looked at the effect of one factor (*stimulus type: sentence or tone sequence*) on another factor (*trial type: same or different*). Regions which can differentiate between these factors are all right sided: the STG, Inferior Frontal Gyrus and Middle Frontal Gyrus. Each of these discriminatory regions show increased activation in tone-sequence, as opposed to sentence, tasks, and during *same *compared to *different *stimuli (Figure 4).

The authors' interpretation of our data is that it best fits with the task dependent hypothesis that the left hemisphere is hemispherically specialized for lexical and short syntactic aspects of pitch whilst the right hemisphere is superior at processing suprasegmental pitch. Subjects' reports place tone sequence and *same *trials as being more difficult and the interaction analysis of activation in the right inferior frontal gyrus (Figure 4) shows increasing activation for these: in both instances subjects are processing a larger, full trial, sequence at a suprasegmental level.

In conclusion, our data support the premise that prosodic pitch perception is subserved by the bifrontal and temporal cortices, specifically the Superior Temporal Gyrus, Inferior Frontal Gyrus and Middle Frontal Gyrus, with the degree of hemispheric involvement dependent upon the task. These areas were activated when both tone-sequence and sentence paradigms were used, thus confounding lexical stimuli were removed, though the former preferentially activated the right hemispheric regions, the latter the left. There was a relative increase in activation in the right frontal and temporal cortices during 'same' stimuli tasks, which was deemed to be more demanding, as subjectively reported by subjects, in terms of prosodic comprehension and this, in our opinion, is due to the need to analyse pitch at a broader 'sentence level'. Our data is in agreement with the assertion [40] of hemispheric specialisation fitting with the task dependent hypothesis, which would have predicted the lateralization [63] found in this study.

Language prosody processing is complex and consists of multiple components. Current understanding of it involves several competing theories, neither of which has garnered consistent support. The vast majority of the literature focuses on emotional prosody: further work is needed to provide a more coherent and distinctive conceptualization of linguistic prosodic processing.

## Authors' contributions

**DKT **participated in the study design, recruited participants, participated in fMRI data collection & analysis, interpreted the results and drafted the manuscript. **DKH **contributed to the drafting of the manuscript. **OOD **recruited participants, participated in fMRI data analysis and contributed to the drafting of the manuscript. **PM **participated in fMRI data collection and interpretation of the results. **KM **participated in the study design and coordination. **LCL **participated in fMRI analysis and contributed to the drafting of the manuscript. **ED **participated in fMRI analysis. **SSS **conceived the study and participated in its design and coordination. All authors read and approved the final manuscript.
